# The Games for Older Adults Active Life (GOAL) Project for People With Mild Cognitive Impairment and Vascular Cognitive Impairment: A Study Protocol for a Randomized Controlled Trial

**DOI:** 10.3389/fneur.2018.01040

**Published:** 2019-01-11

**Authors:** Laura Fabbri, Irene Eleonora Mosca, Filippo Gerli, Leonardo Martini, Silvia Pancani, Giulia Lucidi, Federica Savazzi, Francesca Baglio, Federica Vannetti, Claudio Macchi, The GOAL Working Group, Sandro Sorbi

**Affiliations:** Author Affiliations: Università degli Studi di Firenze, NEUROFARBA, Firenze, Italy; Consorzio di Bioingegneria e Informatica medica - CBIM, Pavia, Italy.; IRCCS Fondazione Don Carlo Gnocchi, Milan, Italy

**Keywords:** mild cognitive impairment, vascular cognitive impairment, cognitive training, tele-rehabilitation, web application

## Abstract

**Background:** People living with Mild Cognitive Impairment (MCI) and Vascular Cognitive Impairment (VCI) are persons who do not fulfill a diagnosis of dementia, but who have a high risk of progressing to a dementia disorder. The most recent guidelines to counteract cognitive decline in MCI/VCI subjects suggest a multidimensional and multi-domain interventions combining cognitive, physical, and social activities. The purpose of this study is to test an innovative service that provides a multi-dimensional tele-rehabilitation program through a user-friendly web application. The latter has been developed through a participatory design involving MCI specialists, patients, and their caregivers. Particularly, the proposed tele-rehabilitation program includes cognitive, physical, and caregiver-supported social activities. The goal is to promote and preserve an active life style and counteract cognitive decline in people living with MCI/VCI.

**Methods:**The study is a randomized controlled trial. Sixty subjects will be randomly assigned to the experimental group, who will receive the tele-rehabilitation program, or the control group, who will not receive any treatment. The trial protocol comprises three steps of assessment for the experimental group: at the baseline (T_0), after tele-rehabilitation program (T_1) and at follow-up after 12-months (T_2). Differently, the control group will be assessed twice: at the baseline and at 12-months follow-up. Both the experimental and the control group will be assessed with a multidimensional evaluation battery, including cognitive functioning, behavioral, functional, and quality of life measures. The tele-rehabilitation program lasts 8 weeks and includes cognitive exercises 3 days a week, physical activities 2 days a week, and social activities once a week. In addition, group will be given an actigraph (GENEActiv, Activisinghts Ltd., Cambridgshire, UK) to track physical and sleep activity.

**Discussion:**Results of this study will inform on the efficacy of the proposed tele-rehabilitation to prevent or delay further cognitive decline in MCI/VCI subjects. The expected outcome is to counteract cognitive decline and improve both physical functioning and quality of life.

**Ethics and Dissemination:**The study is approved by the Local Ethics Committee and registered in https://clinicaltrials.gov (NCT03383549). Dissemination will include submission to a peer-reviewed journal, patients, and healthcare magazines and congress presentations.

**Trial Registration:** ClinicalTrials.gov ID: NCT03383549 (registration date: 26/dec/2017)

**Trial Funding:** Bando FAS Salute 2014 Regione Toscana

**Version Identifier:** ver 5—16/11/2018

## Introduction

Cognitive aging can be successful or lead to impairment depending on the interplay of a manifold of influencing factors. Among these factors, we can count genetic markers, cardiovascular status, neural functional plasticity mechanisms, as well as social, cognitive, and psychological status (1). Within unsuccessful aging paths, the balance between these factors orients toward Mild Cognitive Impairment (MCI) or Vascular Cognitive Impairment (VCI). Mild cognitive impairment (MCI) is a syndrome defined as a cognitive decline which is greater than expected for individual's age and education level but that does not interfere notably with activities of daily life (2). Since his definition in 1999 (3), MCI has been considered the transitional status between normal aging and the diagnosis of a mild dementia (4). MCI is consistently shown to have a high risk of progression to dementia (2, 5) and a pronounced risk of disability and loss of autonomy (6, 7). In addition, cerebrovascular disease that causes brain infarctions becomes more common with advancing age and represents a risk factor for the further development of vascular dementia (8, 9). The construct of VCI, a high-risk phenotype for the development of vascular dementia, has been introduced to capture the entire spectrum of cognitive disorders associated with all forms of cerebral vascular brain injury. There is evidence that about one third of dementia cases globally are attributable to potentially modifiable risk factors that include different daily habits (10, 11). Furthermore, in the study of successful aging compared to the pathological one, the fundamental role that the environment plays in counteracting cognitive decline through the activation of neuro-plasticity mechanisms for the recovery of alternative pathways has been demonstrated (12). Within a holistic framework, the adoption of an appropriate habits alongside cognitive training and enhancement activities can therefore activate brain compensation mechanisms to tackle the physiological and pathological neurodegeneration processes of the elderly, keeping the cognitive level high. On the one hand, there is rising evidence that cognitive training programs are effective in improving or at least maintaining cognitive performance and slowing the progression from MCI or VCI to mild dementia (13, 14). On the other hand, the importance of an intervention that takes into account, beside cognition, also of motor aspects in pre-clinical conditions and in the early stages of dementia has been highlighted (15). To maintain physical abilities and counteract the damage caused by the general physiological decline in the elderly, the adapted physical activity (APA) program has been recognized as an effective tool (16). Furthermore, from a holistic point of view, although social stimulation is often neglected in interventions, recent studies observed that being engaged in social activities represents a protective factor against cognitive decline (17). Therefore, promoting support from the caregivers in social activity participation has been recommended to retain the present abilities and sense of well-being in the persons with cognitive impairment (18). In line with this perspective, the most recent guidelines for the management of MCI/VCI suggest the importance of promoting multidimensional and multi-domain interventions. A multi-dimensional training program integrates different activities, such as the physical and the cognitive one. In addition, the latter could be multi-domains, including exercises that stimulate multiple cognitive domains (i.e., memory, attention, processing speed) (3, 7, 19). Finally, there is evidence that mental, physical, and social stimulation activities equally contribute to decrease dementia risk (20). How to include these activities in a feasible and sustainable rehabilitation service and how to deliver the latter to people living with MCI/VCI, still remains a debated issue. The advancement in technology has enhanced the possibility to provide remote tele-rehabilitation services through low-cost devices, that can ensure continuity of care and can easily reach people living in geographical remote areas. In fact, this kind of services supplies distant support, information exchange between patients and their clinical providers and promotes the administration of multi-dimensional activity programs (21, 22). The lack of familiarity with the technologic devices may constitute a limit to the implementation of technology enhanced services to supply a home-based tele-rehabilitation in people with advanced age or early cognitive impairment (23). For this reason, the development of an accessible service represents a design priority. The use of a participatory co-design process that follows an iterative development model might help in overcoming this limitation (24). The main aim of the proposed trial is to test the performance of a multi-dimensional tele-rehabilitation customized program, which contents are available by a user-friendly web application developed through a participatory design (involving patients, clinicians, technicians, and caregivers). The presented study would investigate whether subjects living with MCI/VCI condition participating in the tele-rehabilitation program will show improvements in cognition, quality of life, adherence, and engagement in the program with respect to subjects living with MCI/VCI condition of the control group.

## Methods and Analysis

### Study Design

**Figure 1:**
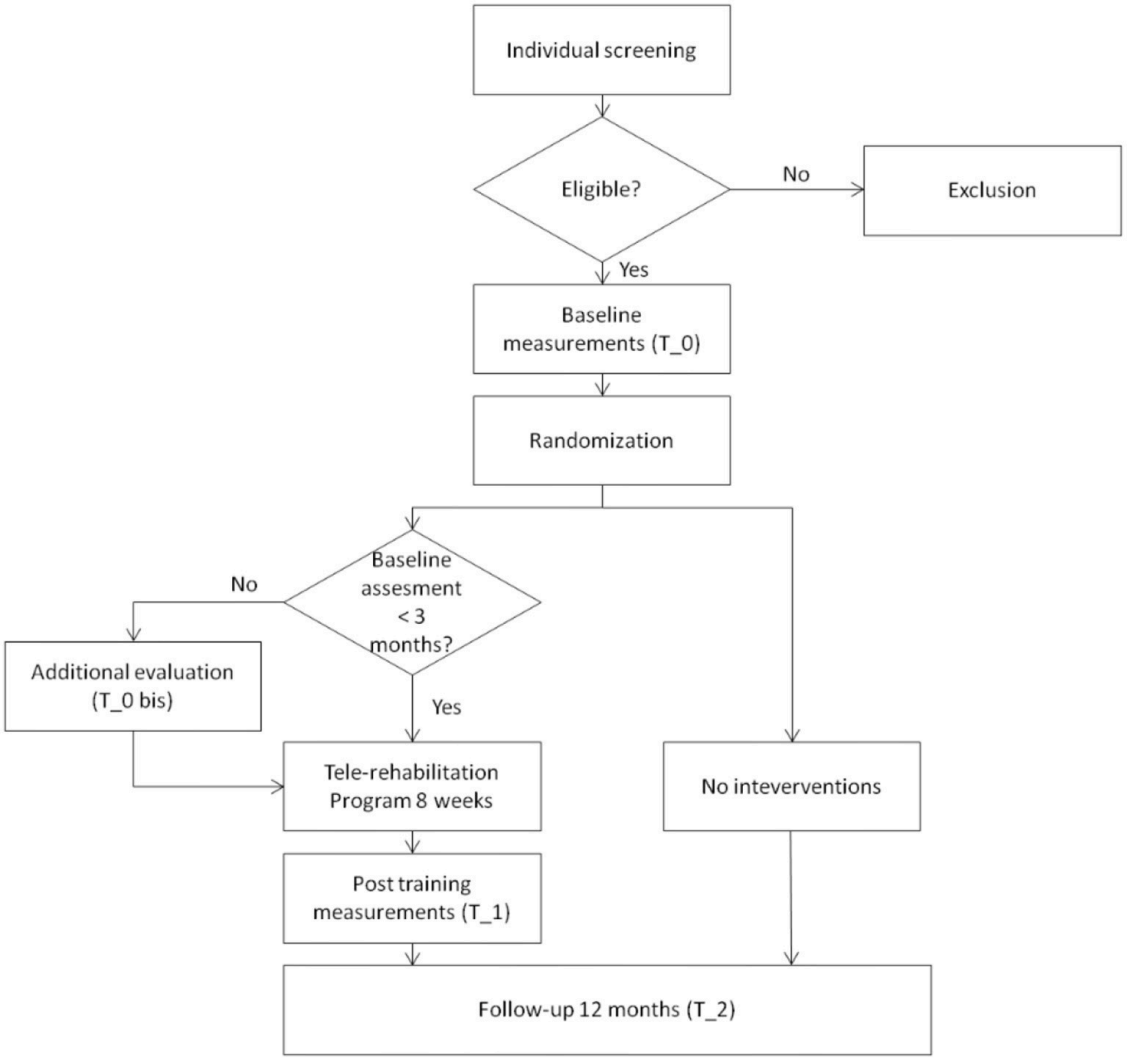
CONSORT flow diagram. Consolidated Standards of Reporting Trials (CONSORT) flow diagram for enrollment and randomization GOAL study.

The proposed study is a randomized controlled clinical trial. A Consolidated Standards of Reporting Trials (CONSORT) flow diagram for enrollment and randomization in the GOAL study is showed in Figure 1. Subjects that, at the first screening will be deemed as eligible, according to the inclusion criteria, will undergo a baseline assessment (T_0). During the baseline a general cognitive and physical evaluation will be performed by using the scales, tests and questionnaires reported in the Participant's evaluation sub-paragraph. Participants will be then randomly assigned, through the use of a computer algorithm (http://www.graphpad.com/quikcalcs/randMenu/) to the control or treatment group. The tele-rehabilitation program will comprise the performing of cognitive exercises 3 days a week, APA activities 2 days a week, and social activities once a week. In addition, treatment group will be given an actigraph (GENEActiv, Activisinghts Ltd., Cambridgshire, UK) to track physical and sleep activity. Those subjects will undergo a post-training assessment immediately at the end of the tele-rehabilitation program (T_1) and after 12 months (T_2). Participants belonging to the control group will be evaluated only at T_2.

### Eligibility Criteria/Participants

Participants will be recruited from the Memory Clinic of University Hospital of Careggi (Florence, Italy), and the Don Carlo Gnocchi Foundation (Florence, Italy). Inclusion criteria will be the following: (a) diagnosis of MCI due to Alzheimer's Disease (AD), according to “Research Clinical Criteria” (25) or diagnosis of Vascular cognitive impairment (VCI) according to the harmonization standards from National Institute for Neurological Disorders and Stroke and the Canadian Stroke Network (8) (Table 1); (b) Mini Mental State Examination (MMSE) ≥24 (26); (c) aged 65–80 years old; (d) school attendance ≥3 years (to avoid both cultural bias and limits due to the neuropsychological instruments used); (e) right-handed according to the Edinburgh Scale (27). Exclusion criteria will be: (a) severe auditory and/or visual loss; (b) severe behavioral/psychiatric disturbances; (c) actual or anamnestic substance abuse disorder; (d) cognitive disturbances secondary to an acute or general medical disorders (e) stable medication of the following pharmacological treatments: cholinesterase inhibitor, memantine, antidepressant, or antipsychotic drugs.

**Table 1:** Criteria for MCI/VCI classification.

**MCI due to AD**	**VCI**
•Hachinski ischemic score ≤ 4 •Exclusion of other dementia-causes with cerebral TC or MRI •Fazekas score < 2 •Biomarkers of neuronal injury (Rate of brain and hippocampal atrophy, FDG-PET imaging or CSF tau/phosphorylated-tau) OR •Biomarker of β amyloid deposition (CSF Ab42 ore PET amyloid imaging) compatible with MCI due to AD	•Hachinski ischemic score >4 •Fazekas score ≥2 •MRI or TC evidence of (one or more): - Multiple white matter lesions compatible with small vessel disease - Lacunar state - One or more ischemic lesions in strategic areas

### Participants' Evaluation

Participants will be assessed through an extensive evaluation including cognitive functioning, behavioral, functional and perceived quality of life measures with the following tests (Table 2):

- The Montreal cognitive assessment [MoCA; (28)], which represents a sensitive tool in persons with MCI for a general cognitive assessment;
- The Digit span and the Corsi span tasks (backward and forward) (29), to assess both verbal and visuo-spatial short-term memory and working memory;
- The Free and Cued Selective Reminding Test [FCSRT; (30)] -Delayed Free Recall (DFR) and Immediate Free Recall (IFR) for verbal episodic memory evaluation;
- The Rey Complex Figure Test, copy and delayed recall [RCFT; (31)], to evaluate constructional skills, visuographic memory and some aspects of planning and executive function;
- The modified card sorting Test, short version [MCST; (32)] for the assessment of executive functions;
- The Trail Making Test [TMT; (33)] for the assessment of attention and -executive functions;
- The Stroop test, short version (34), to assess selective attention, cognitive flexibility and sensitivity to interference;
- The Semantic and Fonemic fluencies (35, 36) or the assessment of language skills;
- The Activities of Daily Living Inventory [ADCS/ADL; (37)] to assess patient's performance in the basic and instrumental activities of daily living.
- The center for Epidemiological Studies Depression scale [CES-D; (38)] to evaluate both the presence and the entity of depressive symptoms;
- The 36-Item Short Form Survey [SF-36; (39)] to assess the subjective perceived quality of live;
- The cognitive Reserve Index questionnaire [CRIq; (40)] for the evaluation of the cognitive reserve;
- The Short physical performance battery [SPPB; (41)], to measure functional status and physical performance;
- The Harvard Alumni Activity Survey [HAAS; (42)], to assess physical activity level;
- The International Physical Activity Questionnaire – Short Form [IPAQ; (43)], for health related physical activity evaluation.

**Table 2:** Evaluation battery during the proposed trial.

**Outcome measures**	***T_0***	***T_1***	***T_2***
**PRIMARY OUTCOME:**			
**General cognitive assessment**	✓	✓	✓
•Montreal cognitive assessment (MoCA) (28)			
**Memory**	✓		✓
•The Digit span and the Corsi span tasks (backward and forward) (29)			
•Free and Cued Selective Reminding Test (FCSRT) (30)	✓		✓
•Recall of rey's figure (31)	✓	✓	✓
**Attention and executive functions**	✓		✓
•The modified card sorting Test, short version (MCST) (32)			
•The Trial Making Test (TMT) (33)	✓		✓
•Stroop test, short version (34)	✓	✓	✓
**Visuospatial/constructional ability**	✓	✓	✓
•Copy of rey's figure (31)			
**Language**	✓	✓	✓
•Semantic fluency (35)			
•Fonemic fluency (36)	✓	✓	✓
**SECONDARY OUTCOMES:**			
**Activities of daily living inventory**	✓	✓	✓
•The Activities of Daily Living Inventory (ADCS-ADL) (37)			
**Depression symptoms**	✓	✓	✓
•The center for Epidemiological Studies Depression scale (CES-D) (38)			
**Health status**	✓		✓
•The 36-Item Short Form Survey (SF-36) (39)			
**Cognitive reserve**	✓		
•The cognitive Reserve Index questionnaire (CRIq) (40)			
**Physical activity level**	✓	✓	✓
•The short Physcal performance battery (SPPB) (41)			
•The Harvard Alumni Activity Survey (HAAS) (42)	✓		✓
•The International Physical Activity Questionnaire (IPAQ) (43)	✓		✓

### Tele-Rehabilitation Program

On the basis of literature, the present protocol is designed aiming at affecting patients' on different domains in line with a holistic approach to promote well-being: cognition, motor skills, and social environment. The cognitive module integrates a collection of brain training exercises from BrainHQ, a third-party platform developed by Posit Science, that makes available Serious Games (SGs) for multidomain stimulation. The proposed SGs are adaptive type, i.e., the difficulty varies in relation to the user performance and becomes more demanding through both reductions in stimulus display duration and increases in task complexity. Difficulty is maintained immediately over the user comfort threshold, which according to several studies, efficaciously stimulates the neural plasticity (44–46). Cognitive activities are meant to reinforce attention, brain speed, memory, people skills, intelligence, and navigation. The physical module includes a training program of APA exercises (16), delivered through a guided video. Regular participation in an APA exercise program has been previously associated with improved self-rated health and improved mood (47). The proposed exercises aim to train trunk, upper, and lower limbs and are designed to require only: a chair, a gym-stick, two small bottles of water and a resistance band, to be performed. Each module comprises a warming-up, a strengthening and a stretching session, with a level of difficulty (basic and advanced) adjusted according to participants' training level and physical capabilities (e.g., participants with reduced ability in mobilizing the upper limbs may have a customized program comprising advanced/basic exercises only for the lower limbs and vice versa). Social level is addressed with the involvement of caregivers and patients in some leisure activities. The caregiver module includes suggestions of social activities to be carried out with the caregiver during the weekend, such as watching a movie, gardening, cooking etc. At the beginning and at the end of daily activity, participants are asked to fill a questionnaire on their self-perceived physical/emotional status.

### GOAL-App Architecture

**Figure 2:**
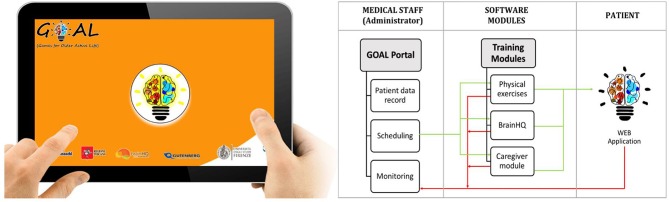
WebApp interface and its functional architecture.

A web-application, named GOAL-App, has been specifically designed to implement a weekly tele-rehabilitation program consisting of combined cognitive, physical and social activities (to be performed with the caregiver), according to the clinician indications. Scheduling and monitoring of activities are accessible by the GOAL-App administrator (Figure 2). Each activity is implemented in the three independent modules on cognition, motor skills and social level. The first realized web-app prototype was developed further through a series of design and feedback loops with MCI specialists, patients living with MCI and VCI and their caregivers. Thanks to this participatory design the following critical aspects emerged and were addressed:

- The need for a full screen interface with a few number of large buttons;
- The possibility to pause the physical module to take breaks during the exercises, if needed;
- The need for a user-manual and a Support Service Center for technical problems.

Patients, provided with a tablet, will access the final version of the tele-rehabilitation program through the GOAL-App. Before the beginning of the treatment, participants will be trained to use the tablet autonomously at their home. During the training sessions participants, with the support of their caregivers, will go through each module of the program, at least once, until they feel familiar with the use of the device. A mobile wifi router will be provided in case participants don't have access to internet connection. In addition, they will be given a kit, made up of a stick and a resistance band, to perform the physical exercises at home.

### Outcomes Measures

As the aim of this program will be to involve the participants in multidimensional activities to counteract cognitive decline, the primary outcome of this study will be the changes of cognitive domains evaluated (such as general cognitive assessment, memory, attention, and executive functions, visuospatial/constructional ability, and language) of the whole sample through a comprehensive neuropsychological assessment at T_0 and at T_2 (see Table 2 for a detailed list of the used neuropsychological tools). Together with the cognitive level, the following secondary outcomes will be evaluated in the whole sample at T_0 and T_2: independence, presence of depressive symptoms, quality of life and physical activity level through scales and actigraphic data (see Table 2 for the used questionnaires). In addition, the primary and secondary outcomes will be evaluated in the treatment group before and after the tele-rehabilitation program using a reduced battery of tests (see Table 2).

### Sample Size

Sample size has been estimated based on previous multicenter controlled studies (21, 48). Under the assumption of normal distribution of the primary outcome scores and considering an α level of 0.05, a sample size of 60 subjects resulted in a power >70% and was then chosen for this trial.

### Data Collection

Assessments will take place at the Don Carlo Gnocchi Foundation (Florence, Italy). All assessors will receive proper instructions and guidance regarding all outcome parameters and assessments that will be taken. A reminder for each visit will be given to all patients to diminish retention and incomplete follow-up.

### Data Management

Study data will be recorded in an access database. All participants will be registered with an identification code with a random order. Source data will include the original documents relating to the study, as well as the medical treatment and medical history of participant. The database will be kept current to reflect subject status at each phase during the course of the study.

### Statistical Analyses

Statistical analysis on outcome measures will be conducted using SPSS version 24 (SPSS Inc, Chicago, IL). Baseline differences between groups will be tested by the independent-samples *t*-test for parametric data and the Mann-Whitney *U*-test for non-parametric data. According to data normality, a statistical test for independent samples will be performed to look at significant changes in primary and secondary outcome measures between control and treatment groups. Moreover, a statistical test for paired sample will be used to investigate the presence of significant changes in the treatment group cognitive and physical parameters before and after the tele-rehabilitation program. An alpha level of ≤ 0.05 will be considered as significant.

## Discussion And Conclusions

In the context of cognitive decline, technologies have been increasingly conceived as a support for patients, their caregivers and the clinicians. The presented trial is claiming to provide MCI/VCI subjects together with their caregivers with an innovative customized tele-rehabilitation program, which contents are available by a user-friendly web application. However, the lack of familiarity with the technologic devices may constitute a limit to the implementation of technology enhanced services to supply a home-based tele-rehabilitation in people with advanced age or early cognitive impairment (23). For this reason, the use of a participatory co-design process that follows an iterative development model might help in overcoming this limitation. The following challenges to program implementation were successfully managed:

- Define the program contents most likely to offer a multi-domain and multi-dimensional stimulation;
- Develop a platform to enable the participants self-administration of the program contents at home;
- Create a user-friendly interface platform to ease a possible lack of familiarity with technology.

The first challenge has been addressed by creating a weekly program that combines cognitive and physical, similar to what has been proposed by Realdon et al. (21). Differently from their project, the physical activity proposed in this program is based on APA exercises and customized to the participant's training level and physical capability. In addition, this program promotes the participation of the subjects in social activities to be conducted with the support of the caregiver. The choice to include in the training program social activities originates from evidence in the literature showing the importance of social stimulation as protective factor against major cognitive decline (17, 49). The second challenge has been met by creating a web-app and providing participants with a tablet and an internet connection, if needed. The possibility to perform the exercises at home, will likely enhance participation in the program. Furthermore, the proposed tele-rehabilitation program leads to a significant reduction of costs compared to previously proposed trainings (50, 51), where participants attended a clinic and were supervised by medical staff. The third challenge has been dealt through a participatory co-design process, used to develop a platform shaped on the basis of user needs. The possible limitations of this study may stem from the restricted numbers of participants enrolled. A potential pitfall could be a drop-out of MCI, therefore the sample size will be increased. Data collection may be impacted by the adherence to the program, especially considering the age of participants, their compliance to the treatment and their medical condition. The monitoring of activity by the administrator is among the several strategies expected to help solve the possible problem of the adherence. However, a high adherence is expected due to the possibility to carry this out at home. To protect sensitive data that will be stored and processed through the web-application, all participants will be registered with an identification code with a random order. Another limitation could be a potential learning effect of neuropsychological instruments due to the timing of the project. This could be avoided by using parallel versions when possible and by a well-structured time scheduling. Some symptoms of apathy, a predictive factor of AD progression (52, 53), are indirectly tested by registering daytime motor activity with actigraphic measures (54, 55). Direct specific measures of apathy cannot be used because of administration issues. The next step is to test the proposed program on subjects living with MCI and VCI condition through the presented trial. The expectation is to counteract cognitive decline, improve physical functioning and quality of life.

## Ethics And Dissemination

The study will be carried out in compliance with the protocol approved by the previously mentioned ethical committee. A written informed consent will be given to each participant (Appendix A in Supplementary Material). The trial is registered at https://clinicaltrials.gov, unique identifier NCT03383549 (registration date: 26/dec/2017). Potentially eligible participants will be screened by the study site principal or sub-investigator for the confirmation of a diagnosis of MCI due to AD or VCI, with a MMSE score ≥24 (26), an age between 65 and 80 years old, a school attendance ≥3 years and with a right-dominant hand. Additionally, potential participants will be questioned and their medical record will be checked in regard to the other eligibility criteria. Given eligibility to take part in the study, they will be provided with further details and an informed consent form by one of the study members. The model consent form and other related documentation given to participants (all in Italian) can be obtained by the corresponding author upon request. The study clinicians will be responsible to explain and make sure the protocol is correctly understood by participants. A separate list with patients screened, but who are not enrolled will contain information regarding the number of patients and the reasons for not enrolling. In order to provide an equal and ethic treatment to all participants, the control group will receive the proposed program at a later date. All anonymized data will be stored in a secure database protected by a password. Only the research team will have access to the database. After the statistical analysis of this trial, the data will be published in a peer-reviewed medical journal, thereby adhering to the CONSORT reporting standards (56) and SPIRIT guidelines (57). The results will be disseminated through peer-reviewed publications, presentation at relevant scientific conferences and the general public.

## Ethics Statement

This study was approved by the Ethical Commitee of IRCCS Don Carlo Gnocchi Foundation and is registered as a clinical trial (NCT03383549). Prospective participants will be fully informed of the aims and procedures of the project. A reporting procedure will be in place to ensure that any serious adverse events are reported to the Chief Investigator. Informed consent will be obtained from all participants and their caregivers before the study initation.

## Author Contributions

LF, IEM, GL, FS, FB, FV, and CM developed the original concept of the trial. LF, FS, FB, FV, and CM drafted the original protocol. LF, IEM, FG, LM, SP, GL, FS, FB, FV, GWG, and CM developed the design. LF, GL, FS, FB, GWG, and CM developed the methodology. LF, IEM, FS, FB, FV, and CM developed the analysis plan. LF, IEM, FG, LM, FB, FV, and CM adapted the trial proposal as a protocol paper. LF, IEM, SP, FV, and CM did manuscript writing. All authors reviewed and commented on drafts of the protocol and paper. All authors read and approved the final manuscript.

### Conflict of Interest Statement

The authors declare that the research was conducted in the absence of any commercial or financial relationships that could be construed as a potential conflict of interest.
