# Supplementary material for: The Games for Older Adults Active Life (GOAL) Project for People With Mild Cognitive Impairment and Vascular Cognitive Impairment: A Study Protocol for a Randomized Controlled Trial
Source: Front Neurol. 2019 Jan 11;9:1040. doi: 10.3389/fneur.2018.01040 (PMC6336896; doi:10.3389/fneur.2018.01040)
Supplement: Supplementary file 1 [file Data_Sheet_1.docx]

**Appendix a**

Model consent form


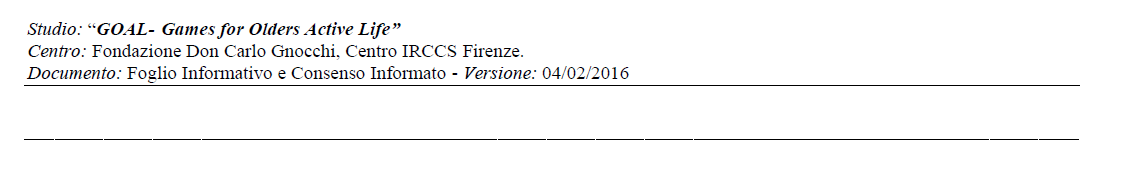


FOGLIO INFORMATIVO E MODULO DI CONSENSO INFORMATO

alla partecipazione al progetto di ricerca

**per i Soggetti ADULTI**

Titolo dello studio: “GOAL- Games for Olders Active Life”

**Egr. Sig. / Gent.le Sig.ra**

La informiamo che stiamo conducendo presso le U.O. di Riabilitazione del Presidio Centro 1 della Fondazione Don Gnocchi ed della Clinica Neurologica 1 dell’Università degli Studi di Firenze un progetto di ricerca dal titolo: **“*Games for Olders Active Life (GOAL)***

Per realizzare questo studio desideriamo avvalerci della collaborazione e della disponibilità di persone come Lei che soddisfano i requisiti idonei all’inserimento nel gruppo di soggetti che intendiamo prendere in considerazione. Per questo motivo Le proponiamo di partecipare allo studio che sarà condotto sotto la responsabilità del Prof. Claudio Macchi.

Prima che Lei decida se accettare o rinunciare, La invitiamo a leggere con attenzione questo documento, qualora Lei desideri avere ulteriori informazioni e chiarimenti potrà rivolgersi alla segreteria organizzativa i cui recapiti sono indicati in fondo al presente documento che Le dedicherà tutto il tempo necessario per chiarire ogni Suo dubbio, fermo restando che Lei potrà rivolgersi in qualsiasi momento anche agli operatori coinvolti nell’esecuzione dello studio.

**Premesse e scopo dello studio**

La Regione Toscana, analogamente alle altre Regioni ed ai paesi Comunitari si trova ad affrontare la problematica delle demenze, spesso conseguente all’invecchiamento della popolazione, stimata (2011) in 86.172 soggetti (prevalenze) e 10.640 nuovi casi (incidenza).

A livello Comunitario (European Innovation Partnership on Active and Healthy Aging) e nazionale (MISE – Programma per il servizio di cura per gli anziani 2013, Presidenza del Consiglio – PIANO NAZIONALE DEMENZE 2014) prioritaria appare, in assenza di approcci farmacologici risolutivi, la tempestività di diagnosi che consenta la messa in opera di interventi clinici e psicosociali che rallentino la progressione.

Stanti i vincoli di bilancio, le nuove tecnologie ICT possono supportare tale priorità, realizzando sistemi informativi innovativi e sostenibili per l’integrazione dei processi di diagnosi, monitoraggio e cura, realizzando nel contempo nuovi strumenti per la valutazione delle capacità cognitive ed il loro potenziamento.

Recenti studi (Anguerra et al, 2013), hanno dimostrato l’evidenza scientifica di training cognitivi tramite software sviluppati in ambiente di realtà virtuale.

Nell’ultimo triennio, il Consorzio di Bioingegneria ed Informatica Medica (CBIM) di Pavia, l'IRCCS Fondazione Istituto Neurologico C. Mondino e l’IRCCS Fondazione Don Carlo Gnocchi, hanno introdotto un modello innovativo, sotto il profilo organizzativo ed operativo per la valutazione funzionale precoce degli stati pre-clinici di demenza, basato su una soluzione ICT che implementa scenari di Realtà Virtuale – Serious Games (SGs) (www.cbim.it/serious-games2/), appositamente sviluppati da uno staff multidisciplinare (neurologi, neuropsicologi, informatici), ad oggi testati su una coorte di oltre 800 soggetti. Nel particolare i SGs realizzati da CBIM costituiscono uno strumento ecologico, progettato come un ambiente di realtà virtuale in 3D, composto da un ambiente domestico in cui il paziente è chiamato ad eseguire compiti vicini alla vita reale con cui vengono valutate le sue funzioni cognitive.

Il progetto GOAL consentirà di disporre di tali strumenti innovativi ed a basso costo, integrati nella piattaforma Habilis della società Signo Motus, che fornirà con analogo approccio strumenti di riabilitazione motoria, in una soluzione tecnologica interoperabile con le strutture sanitarie, i case manager (specialista, MMG, …) ed il domicilio del paziente.

Sotto il profilo clinico, il partenariato, oltre a Fondazione Don Carlo Gnocchi Onlus capofila, che parteciperà con i Centri di Firenze e Fivizzano, vede la partecipazione della Clinica Neurologica 1 dell’Università di Firenze presso l’AOU Careggi, rappresentando così un modello scalabile a livello Regionale.

**Procedure previste dallo studio**

Come prima cosa, se Lei acconsentirà a partecipare a questo Studio, Le sarà chiesto di firmare il qui presente modulo di Consenso Informato che trova in fondo a questa Scheda Informativa.

In caso di partecipazione allo studio Lei effettuerà alcuni test tradizionali di valutazione psicometrica e alcuni test basati sull’esecuzioni di semplici task, sia cognitivi che motori, ambientati in scenari di Realtà Virtuale, detti Serious Games ed Exergames. Eventualmente sarà invitata ad utilizzare un tablet, che Le sarà gratuitamente consegnato per il tempo di durata dello studio, in cui sarà inserita la suite degli stessi Serious Games ed Exergames per effettuare un periodo di training al proprio domicilio.

Tale protocollo di studio non interferirà in alcuno modo con il trattamento di eventuali patologie da cui Lei sia affetto.

**Possibili benefici relativi alla partecipazione allo studio.**

Alla luce dei dati osservati si acquisiranno dati sull'utilità della piattaforma sviluppata nell’ambito del progetto GOAL come strumento di valutazione e monitoraggio dei deficit funzionali nella popolazione con un declino cognitivo lieve ed a rischio di demenza. Si potranno, inoltre, ottenere nuovi strumenti per la gestione clinico-terapeutica dei soggetti con declino cognitivo lieve al domicilio con possibile ricaduta sul SSN per protocolli di intervento non farmacologici nel soggetto a rischio di demenza.

La partecipazione allo studio non implica per il soggetto l’esclusione o la riduzione del trattamento ordinario a cui è sottoposto.

**Possibili rischi / effetti collaterali legati alla partecipazione allo studio.**

La partecipazione a questo studio non comporterà per Lei nessun rischio in quanto non determina variazioni nella gestione del trattamento ordinario, né implica la necessità di eseguire successivamente procedure o interventi aggiuntivi.

**Assicurazione**Data la natura non farmacologica dello studio proposto e la conseguente assenza di rischi aggiuntivi rispetto alla pratica clinica ordinaria, non è necessaria una polizza assicurativa aggiuntiva rispetto a quella già stipulata e tuttora in vigore presso la Fondazione Don Gnocchi per la pratica clinica ordinaria.

**Partecipazione allo studio**

La Sua partecipazione è completamente libera e volontaria.

Se Lei acconsente a partecipare Le sarà chiesto di firmare il Modulo di Consenso Informato, allegato al presente documento, prima che Lei inizi a eseguire la procedura prevista dallo studio.

La firma del modulo allegato è al fine di garantire che Lei abbia ricevuto un’informazione completa e che abbia espresso liberamente la Sua volontà di partecipare; tale firma non implica alcun impegno da parte Sua a proseguire lo studio, non costituisce un vincolo di natura contrattuale, né rappresenta una rinuncia ai diritti che Le spettano.

Nel caso in cui Lei decida di ritirarsi dallo studio, dopo avere inizialmente accettato, potrà interrompere la Sua partecipazione in qualsiasi momento dandone comunicazione al responsabile dello studio senza dover fornire una giustificazione. La scelta di non partecipare, o di ritirarsi dopo l’iniziale accettazione, non comporta l’esclusione o la limitazione delle cure e dell’assistenza che Lei riceve presso le nostre strutture, né alcuna penalizzazione nel Suo rapporto con il personale che La assiste.

Qualora si venisse a conoscenza di nuovi dati o di risultati che possano influenzare la Sua partecipazione allo studio ne sarà tempestivamente informato/a; inoltre, il Responsabile dello studio potrà ritirarLa dallo studio qualora ritenga che tale decisione risponda al Suo migliore interesse.

Sul piano economico la partecipazione allo studio non determina alcun tipo di onere o di spesa aggiuntiva a Suo carico.

Precisiamo che non Le viene richiesto di partecipare a questo studio per ricevere assistenza clinica, o per ottenere beneficio personale di tipo diagnostico, in ogni caso le possibilità di cura di cui dispone il Centro della Fondazione a cui Lei afferisce saranno sempre a Sua disposizione.

La partecipazione a questo studio non comporta alcuna modificazione della normale pratica clinica già seguita: la strategia terapeutica non dipende dalla partecipazione allo studio e non verrà eseguita alcuna procedura supplementare di diagnosi o monitoraggio.

**Trattamento dei dati personali**

*Titolari del trattamento e finalità*

La Fondazione Don Carlo Gnocchi-Onlus, persona giuridica privata nella persona del legale rappresentante pro-tempore, Mons. Angelo Bazzari, con sede legale in P.le R. Morandi, 6 – 20121 Milano, in accordo con le responsabilità previste dalle norme di pratica clinica (D.M. 15.7.1997, D.Lgs. 211/2003, D.Lgs. 200/2007) e di protezione dei dati personali (D.Lgs. 196/2003) nonché dalle disposizioni dell’Autorità Garante per la protezione dei dati personali^[[1]](#footnote-1)^ tratterà in qualità di Titolare i Suoi dati personali e i Suoi dati sensibili relativi al Suo stato di salute, nella misura in cui sono indispensabili in relazione all’obiettivo dello studio ed esclusivamente in funzione della realizzazione dello stesso.

Responsabile del trattamento è stato designato il Dr. Francesco Converti (Direttore del Presidio Centro 1 della Fondazione Don Gnocchi) che ha ricevuto istruzioni scritte anche per le finalità della tutela della riservatezza dei dati sensibili.

*Natura del conferimento dei dati*

Il conferimento dei dati personali sopramenzionati è facoltativo, ma l’eventuale rifiuto, totale o parziale, al conferimento e al trattamento da parte Sua non Le consentirà di partecipare allo studio.

*Natura dei dati e modalità di trattamento*

Tutte le informazioni, personali e cliniche che La riguardano, raccolte durante questo studio sono confidenziali e saranno trattate nel rispetto della normativa vigente sopra richiamata.

I dati raccolti saranno anonimizzati e identificabili tramite un codice solo da parte del responsabile dello studio e del personale che collaborerà nell’esecuzione dello studio.

I dati, trattati mediante strumenti anche elettronici, potranno essere diffusi in forma rigorosamente anonima attraverso riunioni, convegni e pubblicazioni scientifiche; in ogni caso il Suo nome o qualsiasi altro dettaglio idoneo a identificarLa, non saranno divulgati in quanto i dati potranno essere presentati esclusivamente in forma aggregata ovvero secondo modalità che non rendano identificabili i soggetti partecipanti allo studio.

*Esercizio dei diritti*

Lei potrà esercitare i diritti di cui all’art. 7 del D.Lgs. 193/2003 (accedere ai Suoi dati personali, chiederne l’integrazione, l’aggiornamento, la rettifica, la cancellazione, opporsi al trattamento, etc.) rivolgendosi direttamente al Responsabile del trattamento oppure tramite il personale da esso incaricato.

Nel caso in cui Lei si ritiri dallo studio, non saranno più raccolti ulteriori dati che La riguardano, fermo restando l’utilizzo di quelli eventualmente già acquisiti per determinare, senza alterarli, i risultati dello studio.

# Il Protocollo dello Studio a cui Le viene proposto di partecipare, è stato approvato – unitamente al presente documento dal Comitato Etico della Fondazione Don Carlo Gnocchi.

# *Per ulteriori informazioni, chiarimenti e comunicazioni:*

Prof Macchi Claudio, Dott Federica Vannetti, IRCCS, Presidio Centro 1 “Don Carlo Gnocchi” di Firenze, Fondazione Don Carlo Gnocchi ONLUS

Via di Scandicci 269, 50143, Firenze; Tel: 055 73.93.653/909

# *La ringraziamo per la Sua disponibilità e la Sua collaborazione*


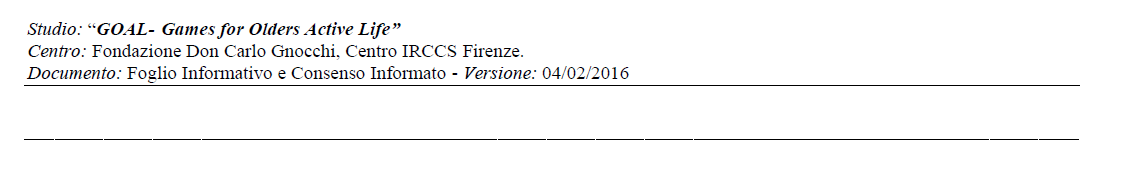


MODULO DI CONSENSO INFORMATO per la partecipazione allo studio:

***“GOAL- Games for Olders Active Life”***

**per i Soggetti ADULTI**

Io sottoscritto/a: __________________________________________________________________________

Cognome e Nome in stampatello del soggetto adulto partecipante.

nato/a a, il: __________________________________________________________________________

*Luogo e data di nascita del soggetto adulto partecipante.*

**dichiaro quanto segue:**

1. ho letto e compreso il foglio informativo di cui questo modulo è parte integrante;
2. ho avuto la possibilità di porre domande e di chiedere spiegazioni al/alla Dr./D.ssa ___________________________________ dal/dalla quale ho ricevuto risposte soddisfacenti;
3. mi sono state illustrate la natura, lo scopo e la durata dello studio, le procedure che saranno seguite, il trattamento previsto per i partecipanti e il tipo di collaborazione che ad essi sarà richiesta;
4. ho compreso che la mia partecipazione allo studio è libera e volontaria e che in qualsiasi momento posso decidere di ritirarmi dallo studio senza essere in alcun modo privato/a delle cure e dell’assistenza di cui ho bisogno e dell’eventuale accesso a nuove prospettive diagnostiche e/o terapeutiche e senza che siano compromessi i miei diritti e il mio rapporto con il medico e con gli operatori sanitari;
5. ai sensi del Decreto Legislativo n.196/2003 e successive modificazioni, acconsento al trattamento dei miei dati personali e sensibili raccolti nell’ambito di questo studio nei termini e nei modi indicati nel presente documento.

**Tutto ciò premesso, nella mia piena capacità di intendere e di volere e senza alcuna forma di condizionamento o coercizione, accetto la proposta di partecipare allo studio descritto nel presente documento.**

Luogo e data: ____________________________ Firma: _____________________________

# Parte riservata all’operatore che ha presentato l’informativa

Io sottoscritto/a Dr/D.ssa ________________________________ *(Cognome e Nome in stampatello)*

**dichiaro:**

1. di avere spiegato alla persona sopraindicata la natura e lo scopo dello studio, nonché le procedure che saranno adottate e il tipo di collaborazione che sarà richiesta;
2. di non avere cercato di influenzare o di costringere in alcun modo la persona sopra indicata per indurla a manifestare il suo consenso alla partecipazione allo studio;
3. di rilasciare alla persona sopraindicata una copia firmata e datata del presente modulo insieme al foglio informativo.

Luogo e data: ____________________________ Firma: _____________________________

1. *Laddove applicabili:* Linee Guida per i trattamenti dei dati personali nell’ambito delle sperimentazioni cliniche dei medicinali (Deliberazione n.52 del 24.07.2008) e Autorizzazione del 27.02.2007 relativa al trattamento dei dati genetici. [↑](#footnote-ref-1)
